# Case report: successful retrieval of an entrapped balloon during balloon pericardiotomy after rupturing

**DOI:** 10.1093/ehjcr/ytag580

**Published:** 2026-07-31

**Authors:** Justin Ka-Ho Wong, John Cheong-Hin Chan, In-Wai Tam, Kwok-Ho Yau, Kin-Lam Tsui

**Affiliations:** Department of Medicine, Pamela Youde Nethersole Eastern Hospital, 3 Lok Man Road, Chai Wan, Hong Kong SAR; Department of Medicine, Pamela Youde Nethersole Eastern Hospital, 3 Lok Man Road, Chai Wan, Hong Kong SAR; Department of Medicine, Pamela Youde Nethersole Eastern Hospital, 3 Lok Man Road, Chai Wan, Hong Kong SAR; Department of Medicine, Pamela Youde Nethersole Eastern Hospital, 3 Lok Man Road, Chai Wan, Hong Kong SAR; Department of Medicine, Pamela Youde Nethersole Eastern Hospital, 3 Lok Man Road, Chai Wan, Hong Kong SAR

**Keywords:** Case report, Malignant pericardial effusion, Percutaneous balloon pericardiotomy, Balloon rupture, Entrapment

## Abstract

**Background:**

Balloon rupture and entrapment during percutaneous balloon pericardiotomy (PBP) is rare and usually requires surgical intervention. However, various bailout techniques can be tried. We describe a case of balloon entrapment in the pericardium and the strategies employed to successfully remove the balloon percutaneously.

**Case summary:**

A 58-year-old woman presented with recurrent malignant pericardial effusion. She refused surgery and opted for PBP. During the procedure, the balloon ruptured and was trapped within the pericardium. The entrapped balloon was secured by snaring onto the proximal end of the balloon. The distal end of the balloon remained trapped across the pericardium as it did not deflate completely after rupturing. Therefore, a needle was inserted under fluoroscopic guidance to decompress the balloon, which enabled its successful removal percutaneously.

**Discussion:**

This is the first reported case of balloon rupture and entrapment during PBP that was successfully removed percutaneously. When balloon rupture and entrapment occur, retrieval can be attempted by using forceps and snares.

## Introduction

Percutaneous balloon pericardiotomy (PBP) is a less invasive alternative to surgery for patients with recurrent malignant pericardial effusion (MPE).^1,2^ The procedure has a high success rate, and complications are uncommon.^1–3^ We present a rare case of balloon rupture and entrapment in the pericardium during PBP that was successfully removed percutaneously, thus avoiding a surgical bailout.

## Summary Figure

**Figure:**
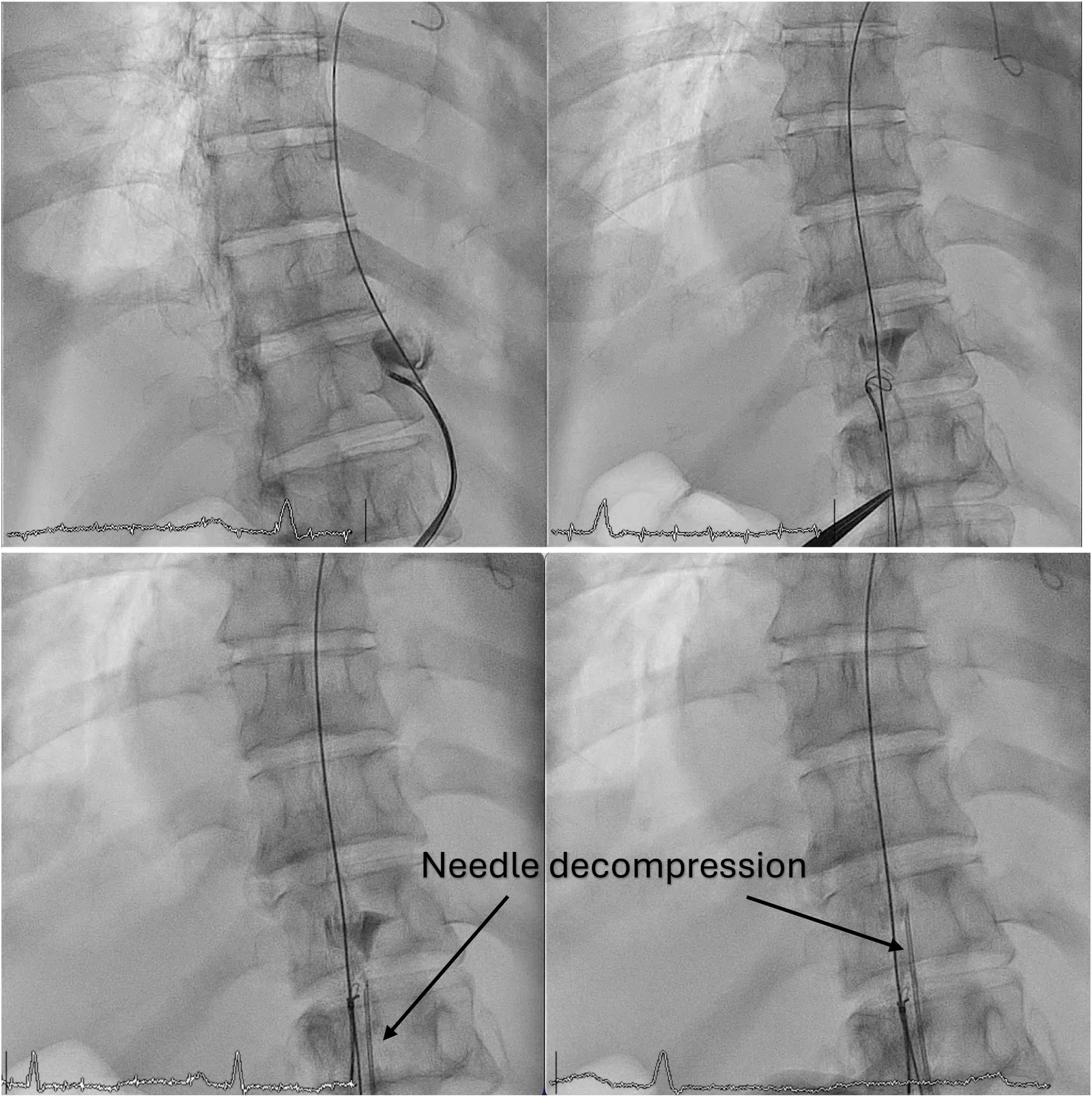


## Case presentation

A 58-year-old female patient with a history of metastatic adenocarcinoma of the lung underwent pericardiocentesis 15 months ago for acute cardiac tamponade. A follow-up echocardiogram 3 months later showed no recurrence of effusion. She was initially on targeted therapy with afatinib, an oral tyrosine kinase inhibitor; however, her cancer continued to progress and, as such, she was referred for palliative care.

Six months later, the patient presented again with acute onset of shortness of breath. On examination, she was tachypneic and tachycardic. Her jugular venous pressure was elevated, and heart sounds were muffled. An echocardiogram showed a large MPE. She underwent another emergency pericardiocentesis via a subxiphoid approach with insertion of a 6-Fr access sheath and a draining pigtail catheter. She was offered the options of either surgical pericardial window or PBP for recurrent MPE. She declined surgical procedure and opted for PBP.

**Figure 1:**
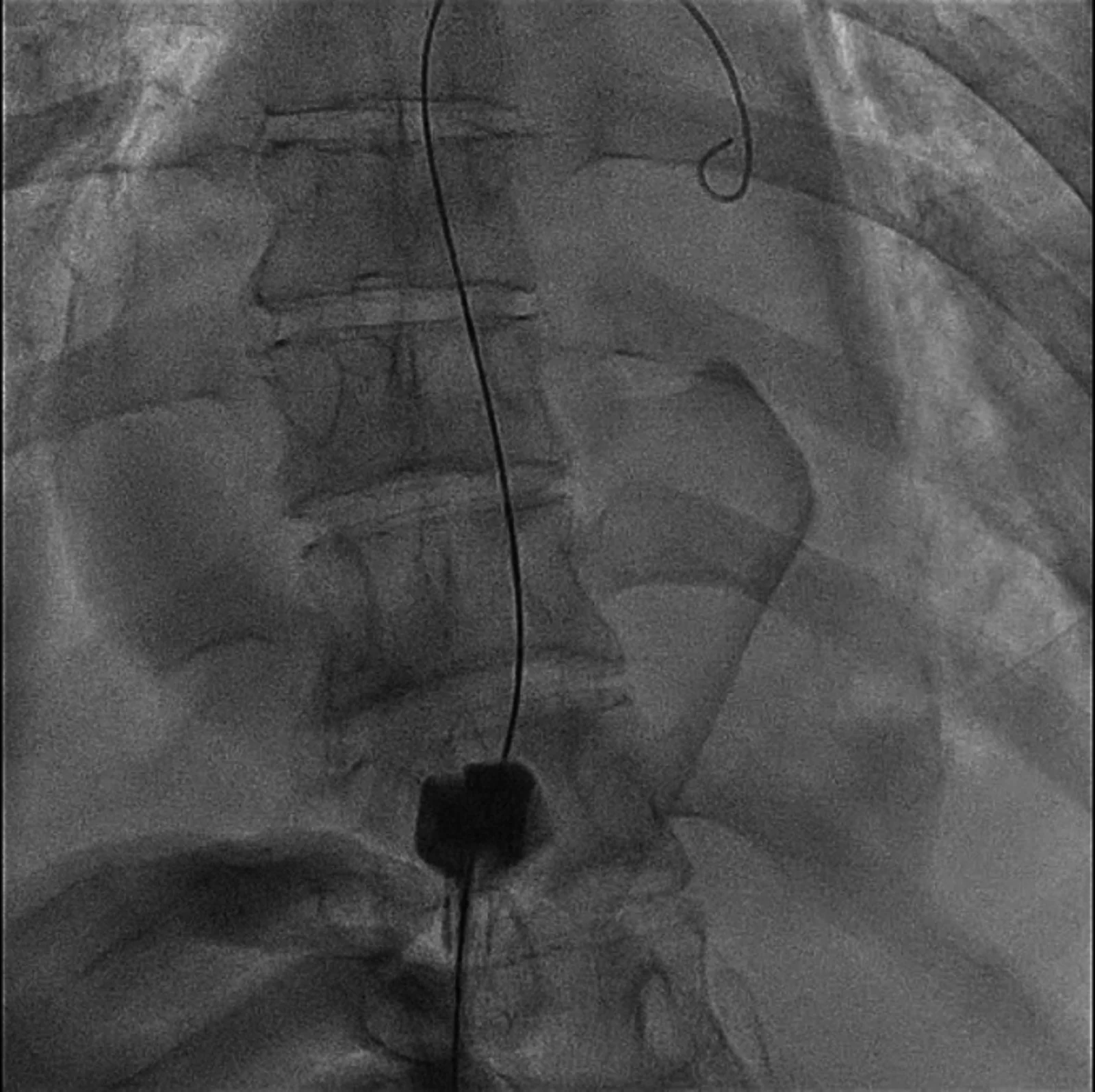
The balloon ruptured during inflation and the distal end of the balloon failed to deflate completely.

PBP was performed under analgesia and light sedation with intravenous boluses of morphine and midazolam. The existing subxiphoid 6-Fr access sheath was exchanged for a 10-Fr sheath and contrast was injected to demarcate the pericardial borders. An Amplatz Extra-Stiff 0.035-inch wire was advanced into the pericardium and an Atlas Gold 20 mm × 40 mm over-the-wire balloon (Becton Dickinson) was positioned across the pericardial border. The balloon failed to dilate the pericardium and repeatedly slipped into the pericardial space. The balloon was switched to an Armada 8.0 mm × 20 mm balloon (Abbott), which successfully dilated the pericardium. The 20 mm × 40 mm long balloon was then reused. During slow inflation, the balloon ruptured at 24 atm, and the distal end of the balloon failed to deflate completely. On further attempt to withdraw the balloon, the balloon became completely separated from the shaft and remained trapped inside the pericardium (*Figure 1*).

**Figure 2:**
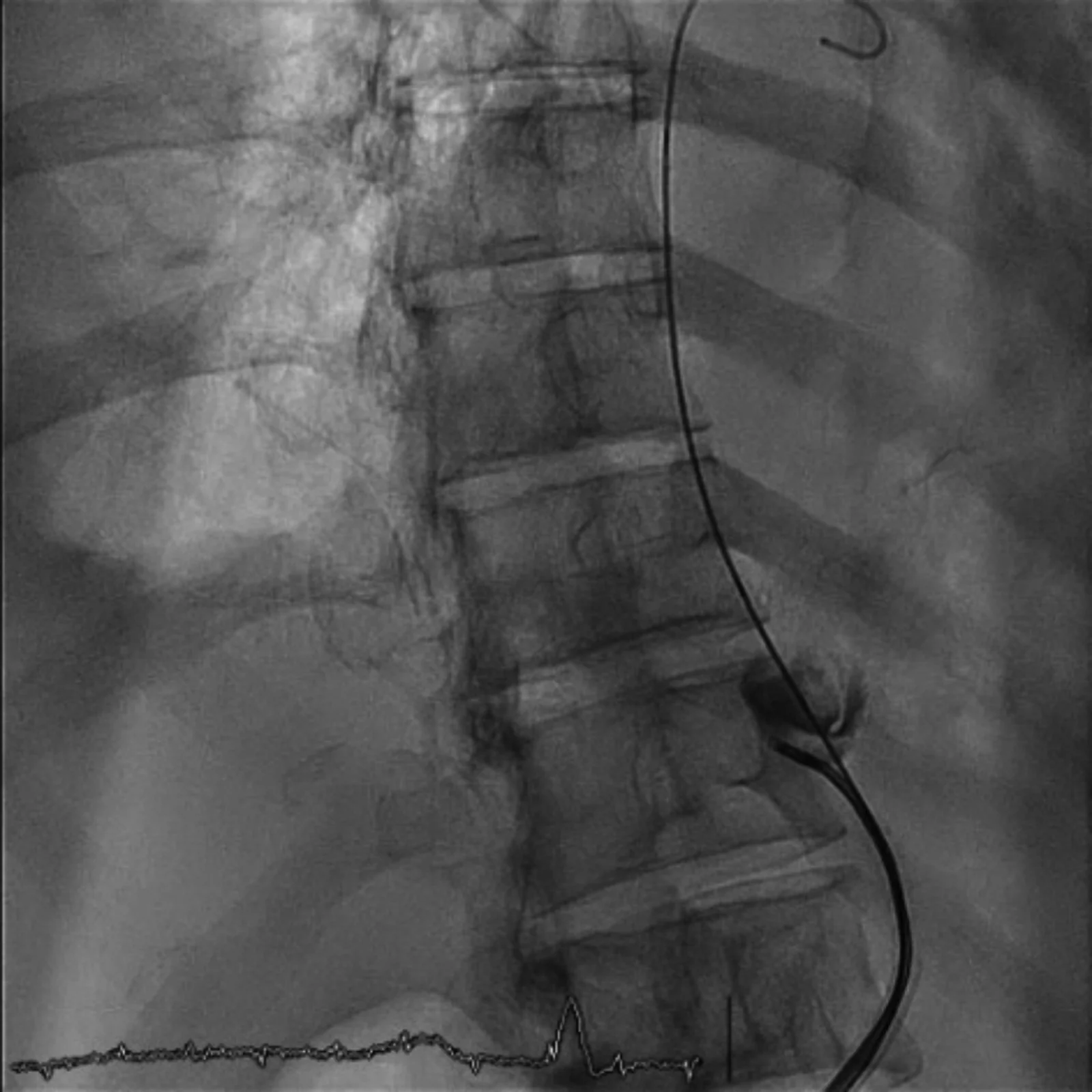
Attempt to use endomyocardial biopsy forceps to retrieve the balloon.

In the first instance, we tried to retrieve the trapped balloon with endomyocardial biopsy forceps. However, the forceps failed to grasp the balloon despite multiple attempts (*Figure 2*).

**Figure 3:**
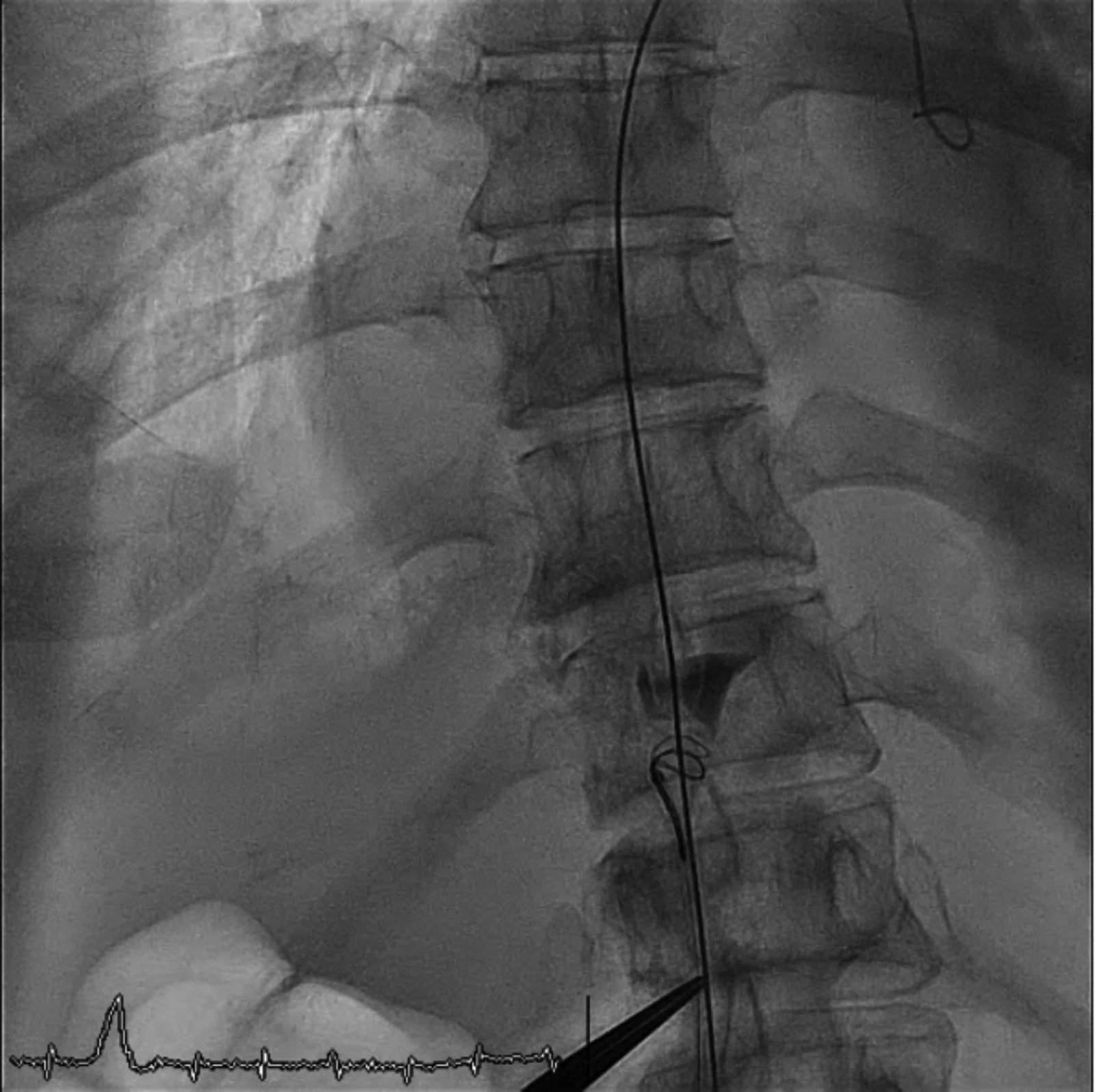
EN snare was able to grasp the proximal end of the balloon.

**Figure 4:**
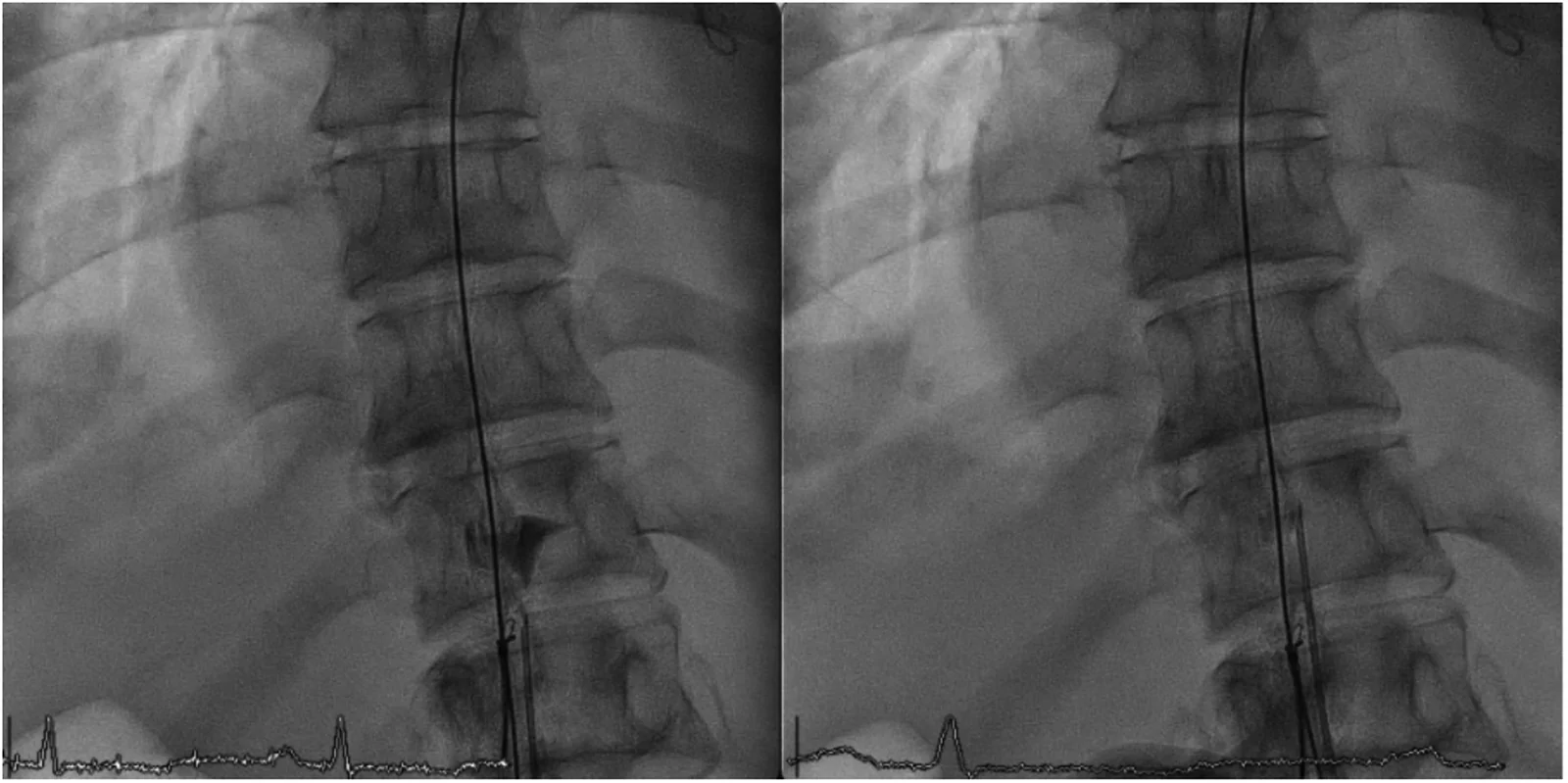
Distal end of the balloon failed to deflate completely, as evidenced by contrast remaining within the balloon tip (left). Needle decompression was performed to deflate the balloon completely (right).

**Figure 5:**
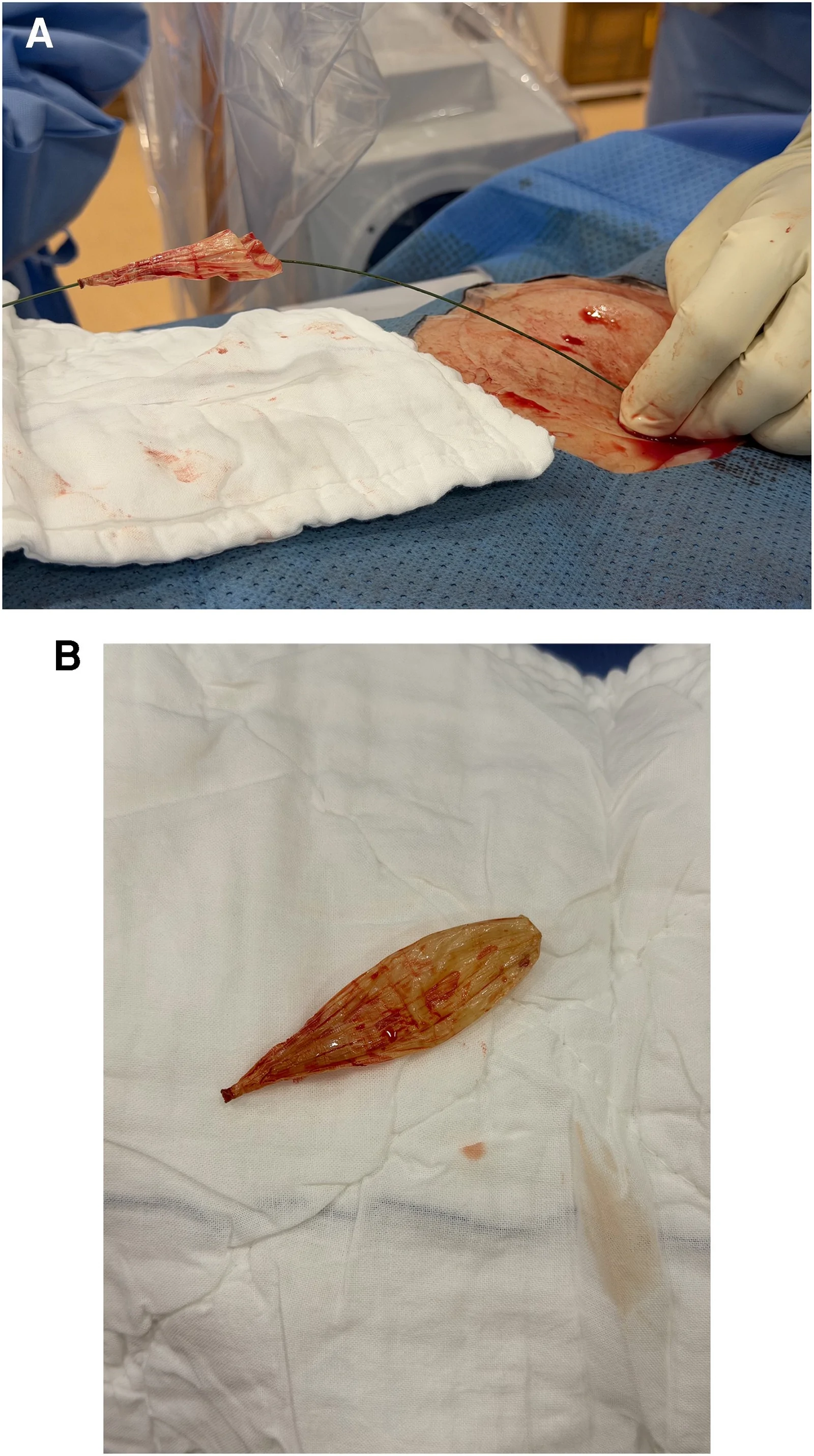
(*A*) The ruptured balloon was successfully removed. (*B*) The balloon was intact, except for a minor tear on its side.

Next, we passed a 7-Fr EN Snare (Merit Medical) along the 0.035-inch guidewire and successfully grasped the proximal end of the balloon (*Figure 3*). However, the distal end of the balloon was trapped across the pericardium and could not be removed from the pericardium as the balloon did not deflate completely after rupturing. Hence, we inserted an 18-gauge needle adjacent to the access sheath, directing the tip towards the residual contrast within the balloon under fluoroscopy to decompress the balloon (*Figure 4*). This enabled the successful removal of the entire balloon through the skin (*Figure 5A*). The ruptured balloon was examined, which only showed a minor tear on its surface but was otherwise intact (*Figure 5B*). We performed repeated contrast injections into the pericardium, which showed successful pericardiotomy with free contrast drainage away from the pericardial space. A CT thorax was performed after the procedure, which confirmed no fragments remained in the pericardium. A pigtail catheter drain was kept *in situ* as a precaution, which was removed on Day 6 after confirming no reaccumulation of MPE, and the patient was subsequently discharged.

At 1-month follow-up, the patient remained well without recurrence of symptoms. A repeat echocardiogram during this period confirmed no re-accumulation of pericardial effusion.

## Discussion

PBP is an increasingly recognized treatment option for recurrent MPE.^1–3^ Although surgical pericardial window remains the standard treatment, many patients with MPE are poor candidates for surgical treatment. Previously, several case registries have demonstrated the successful use of PBP in managing recurrent MPE.^1–3^

More recently, the first randomized controlled trial looking at the efficacy of PBP was also published.^4^ The study demonstrated the superiority of PBP in reducing MPE recurrence when compared with pericardiocentesis at 6 months (12% in the PBP group compared with 60% in the pericardiocentesis group), as well as a lower incidence of cardiac tamponade^4^ (4% in the PBP group vs. 40% in the pericardiocentesis group). In this small study, patients who were assigned to the PBP group underwent the procedure as a first-line intervention, although five patients received routine pericardiocentesis first due to logistical reasons. However, the study was limited by its small number of recruited patients and a relatively short follow-up period of 6 months.

PBP has a high procedural success rate, ranging from 93% to 100%.^1–3^ Serious complications are uncommon; however, tension pneumothorax, myocardial tear, and bleeding from pericardial vessel injury requiring urgent surgery have been reported.^1,3,4^ In our case, the procedure was complicated by balloon entrapment within the pericardial space following balloon rupture. This is the first reported case of balloon rupture during PBP, and thus the incidence rate is unknown. We postulate that the reason the balloon ruptured is due to inflation of the balloon against a thickened, fibrotic pericardium from underlying chronic disease. In fact, a pre-PBP echocardiogram showed many adhesions within the pericardial space, which may increase the difficulty in maintaining the balloon across the fibrotic pericardium. This may inadvertently result in the operator applying incremental traction force on the balloon to prevent it from slipping into the pericardial space. In hindsight, a second (buddy) guidewire may be useful in maintaining the position of the balloon.^3^ The stabilized balloon, together with the buddy wire itself, may facilitate breaking of the fibrotic pericardium without the need to apply excessive traction force on the balloon. If, however, despite using a buddy guidewire and the balloon cannot be stabilized, then such cases may not be suitable for PBP and should be referred for surgery.

## Conclusion

When balloon rupture and entrapment occur, retrieval can be attempted by means such as forceps and snare as in the present case. It is prudent to keep the 0.035-inch guidewire *in situ*, which allows passage of the snare. When the balloon cannot be extracted out of the pericardial space because of the bulkiness of the large balloon, which is not fully deflated, needle insertion guided by the residual contrast in the balloon, followed by aspiration can decompress the balloon, resulting in successful retrieval, which spares the need for a surgical bailout.

## Data Availability

The data underlying this article will be shared on reasonable request to the corresponding author.

## Lead author biography

Associated Consultant (Cardiology)

## Notes

**Consent:** The patient has given written consent for the publication of this case report in compliance with COPE guidelines.
